# LEVEL OF OBSTACLES BETWEEN WORK AND CARE PROVISION AND LIFE SATISFACTION AMONG JAPANESE WORKING FAMILY CAREGIVERS

**DOI:** 10.1093/geroni/igad104.3053

**Published:** 2023-12-21

**Authors:** Ayumi Honda, Yin Liu, Elizabeth Fauth, Sumihisa Honda

**Affiliations:** St Mary’s College, Kurume, Fukuoka, Japan; Utah State University, Logan, Utah, United States; Utah State University, Logan, Utah, United States; Nagasaki University, Nagasaki, Nagasaki, Japan

## Abstract

**Objectives:**

This study aimed to examine whether facing obstacles between work and care provision (work/care obstacles) is a risk factor for life satisfaction and explore whether it moderates the association between care burden and life satisfaction among Japanese working family caregivers.

**Methods:**

This cross-sectional study was conducted on 141 working family caregivers aged less than 65 years who were living with an older care recipient. We fit a multiple logistic regression model to examine the main and moderating effect of work/care obstacles on life satisfaction, in the context of care burdens.

**Results:**

Facing work/care obstacles had a main effect on life satisfaction among employed family caregivers. Furthermore, work/care obstacles exacerbated the association between caregiver burden and life satisfaction. As for the family caregivers who did not experience work/care obstacles, the risk of poor life satisfaction did not differ regardless of number of caregiver burdens. In contrary, the family caregivers who experienced work/care obstacles and had 2 or more caregiver burdens had a poor life satisfaction (odds rations = 5.5, 95% confidence intervals = [1.97, 15.43]) compared with those who had 1 or less caregiver burden.

**Conclusion:**

These findings suggest that obstacles between work and family was a risk factor for poor life satisfaction for employed individuals in Japan caring for an older relative. Mitigating such obstacles may be important for job retention, given the growing number of older adults in Japan. Key words Facing obstacles between work and care provision, family caregiver, life satisfaction, well-being, work–life adaptation

